# Nonautophagic cytoplasmic vacuolation death induction in human PC-3M prostate cancer by curcumin through reactive oxygen species -mediated endoplasmic reticulum stress

**DOI:** 10.1038/srep10420

**Published:** 2015-05-27

**Authors:** Wei-Jiunn Lee, Ming-Hsien Chien, Jyh-Ming Chow, Junn-Liang Chang, Yu-Ching Wen, Yung-Wei Lin, Chao-Wen Cheng, Gi-Ming Lai, Michael Hsiao, Liang-Ming Lee

**Affiliations:** 1 Department of Urology, Wan Fang Hospital, Taipei Medical University, Taipei, Taiwan; 2 Graduate Institute of Clinical Medicine, College of Medicine, Taipei Medical University, Taipei, Taiwan; 3 Department of Medical Education and Research, Wan Fang Hospital, Taipei Medical University, Taipei, Taiwan; 4 Department of Internal Medicine, Wan Fang Hospital, Taipei Medical University, Taipei, Taiwan; 5 Department of Medical Management, Taoyuan Armed Forces General Hospital, Taoyuan County, Taiwan; 6 Biomedical Engineering Department, Ming Chuan University, Taoyuan County, Taiwan; 7 Division of Hematology and Medical Oncology, Department of Internal Medicine, Wan Fang Hospital, Taipei Medical University, Taipei, Taiwan; 8 The Genomics Research Center, Academia Sinica, Taipei, Taiwan

## Abstract

The antiapoptotic and antiautophagic abilities of cancer cells constitute a major challenge for anticancer drug treatment. Strategies for triggering nonapoptotic or nonautophagic cell death may improve therapeutic efficacy against cancer. Curcumin has been reported to exhibit cancer chemopreventive properties. Herein, we report that curcumin induced apoptosis in LNCaP, DU145, and PC-3 cells but triggered extensive cytoplasmic vacuolation in PC-3M cells. Electron microscopic images showed that the vacuoles lacked intracellular organelles and were derived from the endoplasmic reticulum (ER). Moreover, curcumin-induced vacuolation was not reversed by an apoptosis- or autophagy-related inhibitor, suggesting that vacuolation-mediated cell death differs from classical apoptotic and autophagic cell death. Mechanistic investigations revealed that curcumin treatment upregulated the ER stress markers CHOP and Bip/GRP78 and the autophagic marker LC3-II. In addition, curcumin induced ER stress by triggering ROS generation, which was supported by the finding that treating cells with the antioxidant NAC alleviated curcumin-mediated ER stress and vacuolation-mediated death. An *in vivo* PC-3M orthotopic prostate cancer model revealed that curcumin reduced tumor growth by inducing ROS production followed by vacuolation-mediated cell death. Overall, our results indicated that curcumin acts as an inducer of ROS production, which leads to nonapoptotic and nonautophagic cell death via increased ER stress.

Prostate cancer is the leading cause of cancer-related death among men and represents a salient health threat^1^. However, limited treatment options are available for prostate cancer because of its poor response to current chemotherapy and radiotherapy protocols and because metastatic disease frequently develops even after radical prostatectomy^2^. Androgen deprivation therapy remains the principal treatment for patients with locally advanced and metastatic disease. Although most patients initially respond to androgen deprivation therapy, they ultimately progress to a castration-resistant prostate cancer that acquires the ability to evade cell death under androgen-depleted conditions^3^,^4^. Recently, several reports have indicated that prostate cancer resists chemotherapy and androgen deprivation therapy via antiapoptotic or antiautophagic mechanisms^5^,^6^. These mechanisms ultimately underlie the treatment resistance that characterizes prostate cancer and limit the effectiveness of therapeutic strategies. Therefore, developing approaches that trigger nonapoptotic and nonautophagic cell death in cancer cells to overcome the resistance to apoptosis or autophagy will facilitate the effective treatment of prostate cancer.

Several forms of nonapoptotic and nonautophagic cell death, such as oncosis^7^, necroptosis^8^, entosis^9^, anoikis^10^, and programmed necrosis^11^, have been described according to specific cellular and molecular criteria. Importantly, two similar types of nonapoptotic and nonautophagic cell death: cytoplasmic vacuolation death and paraptosis have been described based on the specific formation of cytoplasmic vacuoles^12^,^13^,^14^. These two types of cell death are morphologically characterized by extensive cytoplasmic vacuolation and endoplasmic reticulum (ER) dilatation but do not involve caspase activation or nuclear changes; however, only paraptosis is associated with mitochondrial swelling^12^,^13^,^15^,^16^. In addition, the protein synthesis inhibitor cycloheximide (CHX) has been reported to block cytoplasmic vacuole formation in both cytoplasmic vacuolation-mediated death and paraptosis^12^,^13^,^15^,^17^. Notably, a previous report indicated that increased expression of the autophagic marker LC3-II and the ER stress markers Bip/GRP78 and CHOP or the accumulation of ubiquitinated proteins is observed in cancer cells undergoing cytoplasmic vacuolation-mediated death^15^. Although the molecular mechanisms underlying apoptosis and autophagy have been extensively characterized, the mechanisms underlying cytoplasmic vacuolation-mediated death are less clearly understood.

The ER plays an important role in the processing, folding and export of newly synthesized proteins to the secretory pathway^18^. Under normal conditions, the ER stress response regulates homeostatic mechanisms within the ER. However, intense or persistent ER stress can induce apoptosis. Recent studies have indicated that ER stress may also contribute to caspase-independent cell death, which is characterized by extensive cytoplasmic vacuolation in cancer cells without the involvement of apoptosis or autophagy^12^,^13^. In addition, ER stress can be triggered by various stimuli, such as hyperhomocysteinemia, oxidative stress and the disturbance of Ca^2+^homeostasis^19^,^20^. Excessive production of ROS can lead to oxidative stress, which can interfere with ER function, causing the accumulation of large amounts of unfolded or misfolded proteins and leading to the cellular ER stress response.

Curcumin, a phytopolyphenolic pigment derived from turmeric (*Curcuma longa*), is commonly used as a flavoring agent in food and is nontoxic to humans^21^. Curcumin acts on various molecular targets associated with anticancer, antiangiogenic, and proapoptotic responses. Preclinical studies have demonstrated the anticancer activity of curcumin in various cancer cell lines, including breast, cervical, colon, brain, gastric, hepatic, oral epithelial, ovarian, and pancreatic cancer, as well as leukemia and melanoma cell lines, and curcumin has been used in clinical trials of colon cancer^22^. In this study, we aimed to investigate the novel effects of curcumin on prostate cancer cells and to obtain new insight into the mechanisms underlying these effects. Our results showed that curcumin was able to inhibit the growth of metastatic PC-3M prostate cancer cells in a highly potent manner by inducing the ROS-mediated activation of ER stress and, subsequently, nonapoptotic and nonautophagic cytoplasmic vacuolation-mediated cell death. In addition, an increase in ROS generation and ER stress was observed in curcumin-induced vacuolation-mediated cell death in a PC-3M orthotopic graft model. These findings suggest, for the first time, that curcumin may represent a novel agent for metastatic prostate cancer treatment.

## Results

### Curcumin treatment results in reduced cell viability and altered morphology of prostate cancer cells

**Figure 1:**
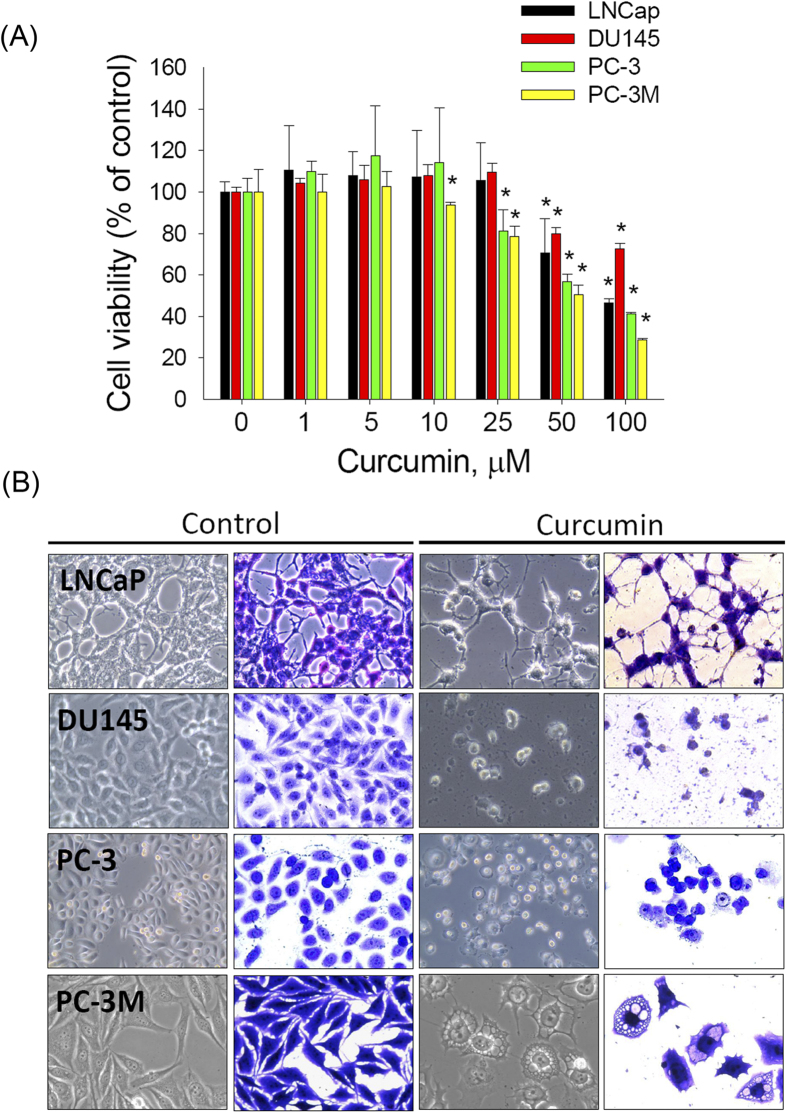
Curcumin treatment results in reduced cell viability and morphological changes in prostate cancer cells. (**A**): The growth inhibitory effect of curcumin on prostate cancer cells. Cells were seeded at 5,000 cells per well in 96-well plates, incubated for 24 h, and treated with vehicle or curcumin (1, 5, 10, 25, 50, or 100 μM) for 48 h; then, the relative cell viability was analyzed by performing the MTS assay. Data are presented as the means ± SD or standard error of 3 independent experiments. * *P* < 0.01. (**B**): The cells were treated with 50 μM curcumin for 48 h, stained or not stained with crystal violet, and photographed under a microscope (40 × magnification).

To explore the anti-prostate-cancer activity of curcumin, the cytotoxic effects of various concentrations of curcumin (approximately 0–100 μM) on 4 prostate cancer cell lines (LNCap, DU145, PC-3, and PC-3M) after treatment for 48 h were analyzed (Fig. 1A). PC-3M is a more highly metastatic cell line than the PC-3 cell line from which it was derived^14^. The MTS assay showed that curcumin reduced cell survival in a dose-dependent manner in all prostate cancer cell lines. Among the 4 examined prostate cancer cell lines, PC-3M cells were found to be the most sensitive to curcumin treatment (Fig. 1A). In addition, the morphological changes in prostate cancer cells were visualized via optic microscopy after the cells were treated with 50 μM curcumin for 48 h. Neurite elongation, a typical phenotype of neuroendocrine differentiation cells, was observed in the LNCaP cells, whereas more detached and shrunken cells were observed in the DU145 and PC-3 cell lines. Furthermore, extensive cytoplasmic vacuolation was observed in the PC-3M cells after curcumin treatment (Fig. 1B). This finding clearly indicated that curcumin potently reduces the cell viability of various prostate cancer cell lines by inducing distinct types of cell death.

### Prostate cancer cell lines exhibit varied responses to curcumin

**Figure 2:**
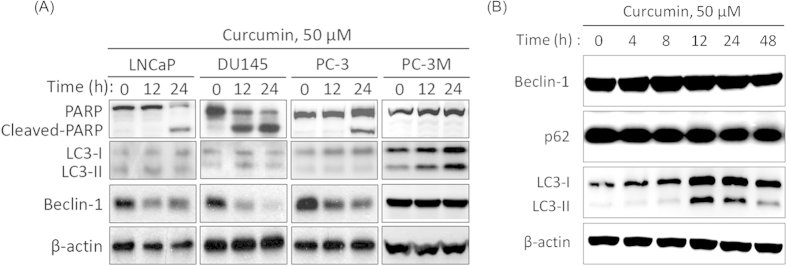
Effect of curcumin on apoptosis- and autophagy-related protein expression in prostate cancer cells. (**A**): Prostate cancer cell lines were incubated in curcumin and harvested at various time points. After treatment with 50 μM curcumin for the indicated intervals, LNCaP, DU145, PC-3, and PC-3M cells were lysed and immunoblotted using antibodies against PARP, Beclin-1, LC3, and β–actin. (**B**): The time course of the protein expression of Beclin-1, p62 and LC3 following treatment with 50 μM curcumin for the indicated intervals based on western blot. β–actin was used as a loading control.

Neurite elongation or cell shrinkage is typically observed in cells undergoing autophagy or apoptosis^23^,^24^. To gain insight into the cell death mechanism, 4 prostate cancer cell lines were treated with 50 μM curcumin for the indicated intervals (0, 12, or 24 h). Kinetic studies after up to 12 h of curcumin treatment showed that the apoptotic marker cleaved PARP was upregulated and that the autophagic marker Beclin-1 was downregulated in the LNCaP, DU145, and PC-3 cells but not in the PC-3M cells. This result indicated that curcumin may induce apoptosis in the aforementioned 3 cell lines but not in the PC-3M cell line (Fig. 2A). In contrast to LNCaP, DU145, and PC-3 cells, treating PC-3M cells with curcumin upregulated the membrane-bound form LC3-II but failed to affect the other autophagic marker, Beclin-1. To further characterize curcumin-induced cytoplasmic vacuolation, PC-3M cells were treated with curcumin for the indicated intervals, and the levels of another autophagic marker, p62, were analyzed. As expected, curcumin treatment induced the persistent expression of LC3-II but did not influence the levels of Beclin-1 or p62 (Fig. 2B). These results suggested that the curcumin-mediated autophagic process was not completed in the PC-3M cells.

### Curcumin induces nonapoptotic and nonautophagic cytoplasmic vacuolation-mediated death in PC-3M cells

**Figure 3:**
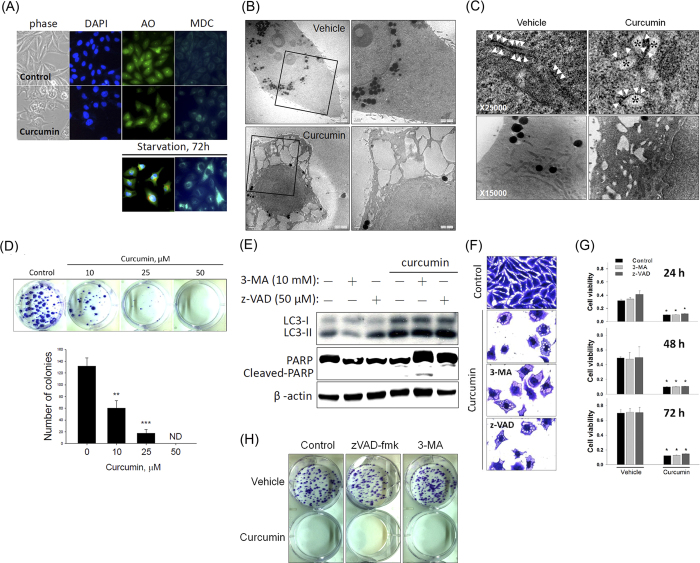
Curcumin induces the nonapoptotic and nonautophagic cytoplasmic vacuolation-mediated death of PC-3M cells. (**A**): Representative images of phase-contrast imaging and DAPI, AO, and MDC staining of PC-3M cells in the presence or absence of 50 μM curcumin for 48 h (upper panel) or under serum starvation for 72 h (lower panel). (**B**): Transmission electron micrograph of untreated (vehicle) and curcumin-treated (50 μM) PC-3M cells for 24 h at × 2500 (left panel) and × 5000 (right panel), showing cytoplasmic vacuoles devoid of intracellular organelles. (**C**): Transmission electron micrograph of untreated (vehicle) and curcumin-treated (50 μM) PC-3M cells at higher magnification (× 25,000), showing the dilation of the rough ER. Ribosomes (arrowheads) are attached to some of these membranes, indicating that these vacuoles (*) were derived from the ER (C, upper panel). After 24 h of curcumin treatment, most mitochondria remained unchanged (C, lower panel). (**D**): The death-inducing effects of curcumin on PC-3M cells were determined by counting the colonies formed. Top: Representative photomicrographs. Bottom: The data are presented as the means ± SD or standard error of 3 independent experiments. ** *P* < 0.01; *** P < 0.001. (**C**–**F**): The *P*C*-*3M cells were pretreated with the autophagy inhibitor 3-MA (10 mM) or the caspase inhibitor z-VAD (50 μM) for 1 h and then further treated with 50 μM curcumin for 48 h. (**E**): Cell extracts were prepared and subjected to western blot for PARP and LC3. (**F**): Cell vacuolation was observed via crystal violet staining. (**G**): Cell viability was assessed by performing the MTS assay. (**H**): Representative photomicrographs of the colony-forming assay.

PC-3M cells exhibited greater sensitivity to curcumin-induced growth inhibition than did LNCaP, DU145, and PC-3 cells; however, they also produced cytoplasmic vacuoles. This observed discordance in cell viability and morphological changes among these cell types may be attributed to the different modes of curcumin-mediated cell death that are operational in the PC-3M cells. To further investigate whether the curcumin-induced cytoplasmic vacuolation-mediated death of PC-3M cells was distinct from apoptotic or autophagic cell death, we first measured the changes in nuclear morphology and autophagic vacuole formation by performing DAPI, acridine orange (AO), and monodansylcadaverine (MDC) staining. Figure 3A shows that treating PC-3M cells with curcumin induced extensive cytoplasmic vacuolation with neither nuclear changes (apoptotic body formation) nor the formation of MDC-labeled fluorescent dots and acidic vesicular organelles (red fluorescence). Serum-starved PC-3M cells were used as a positive control for cells undergoing autophagy (Fig. 3A, lower panel). Transmission electron microscopy (TEM) of PC-3M cells after a 24-h treatment with 50 μM curcumin was analyzed to further characterize vacuole formation. Under electron microscopy, curcumin-induced large cytoplasmic vacuoles appeared clear and lacked any detectable cytoplasmic material and without plasma membrane alteration (Fig. 3B). Furthermore, we observed that the nuclei were intact, thus ruling out autophagy and apoptosis as the mode of cell death (Fig. 3B). Moreover, ribosomes were aligned with the rough ER in untreated cells. Notably, in the curcumin-treated cells, the vacuoles were surrounded by membranes, some of which were decorated by ribosomes (Fig. 3C, upper panel), indicating that the intracellular vacuoles were derived from the rough ER. Although we observed the dilation of the cristae membranes, most mitochondria remained unchanged even after a 24-h treatment with curcumin (Fig. 3C, lower panel), indicating that paraptosis is not the major cause of curcumin-mediated cytoplasmic vacuolation in PC-3M cells.

We then performed a clonogenic assay to determine the long-term growth inhibitory effect of curcumin. The PC-3M cells were treated with various concentrations of curcumin (0, 10, 25, or 50 μM) for 48 h and then continuously cultured in fresh media for the next 14 d, at which point colony formation was measured. The number of surviving colonies was significantly reduced by curcumin in a dose-dependent manner (Fig. 3D). We further tested the effects of autophagy and apoptosis inhibitors on the curcumin-mediated increases in LC3-II expression, cytoplasmic vacuolation, and cell death, as well as on the decrease in colony formation. Figure 3C–F shows that the curcumin-induced increases in LC3-II expression (Fig. 3E), cytoplasmic vacuolation (Fig. 3F), and cell death (Fig. 3G) and the decrease in colony formation (Fig. 3H) were not significantly affected by treatment with the autophagy inhibitor 3-methyladenine (3-MA) or the apoptosis inhibitor z-VAD. Overall, these data indicated that the curcumin-induced irreversible cytoplasmic vacuolation-mediated death of PC-3M cells does not involve the apoptotic or autophagic cell death pathway.

### Curcumin induces cytoplasmic vacuolation-mediated cell death by triggering ER stress

**Figure 4:**
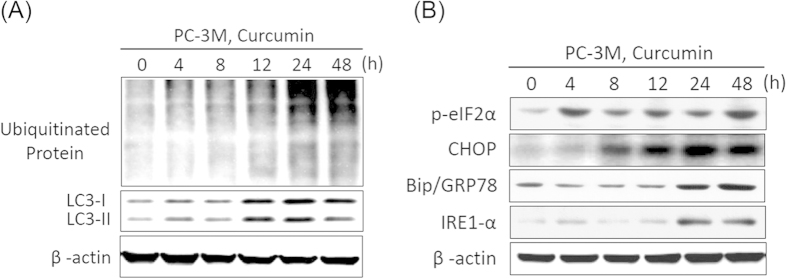
Curcumin induces cytoplasmic vacuolation-mediated cell death by increasing ER stress. PC-3M cells were treated with 50 μM curcumin for the indicated intervals, and the expression of LC3, polyubiquitinated proteins (**A**), and the ER stress markers phospho-eIF2α, CHOP, Bip/GRP78, and IRE1-α (**B**) were analyzed via western blot. Equal protein loading was confirmed in the western blot by probing for β-actin.

The marked dilation of ER cisternae caused by curcumin treatment suggested that swelling of the ER compartment might occur as a result of ER stress. Indeed, a previous report indicated that ER stress and protein ubiquitination are characteristics of cytoplasmic vacuolation-mediated cell death^13^,^17^. Thus, we examined whether changes in ER stress and protein ubiquitination are involved in curcumin-induced cytoplasmic vacuolation. Western blot analysis showed that curcumin enhanced LC3 expression and was accompanied by increased polyubiquitinated protein levels (Fig. 4A). This result indicated activation of the unfolded protein response (UPR) pathway after curcumin treatment. Furthermore, we examined whether ER-stress-associated proteins are modulated by curcumin in PC-3M cells. The expression of the ER-stress-associated proteins phospho-eIF2α, CHOP, Bip/GRP78, and IRE1-α was significantly induced by curcumin (Fig. 4B). These results suggested that ER stress may play a crucial role in the curcumin-induced vacuolation-mediated death of PC-3M cells.

### Curcumin-induced cytoplasmic vacuolation requires new protein synthesis and is prevented by LC3 knockdown

**Figure 5:**
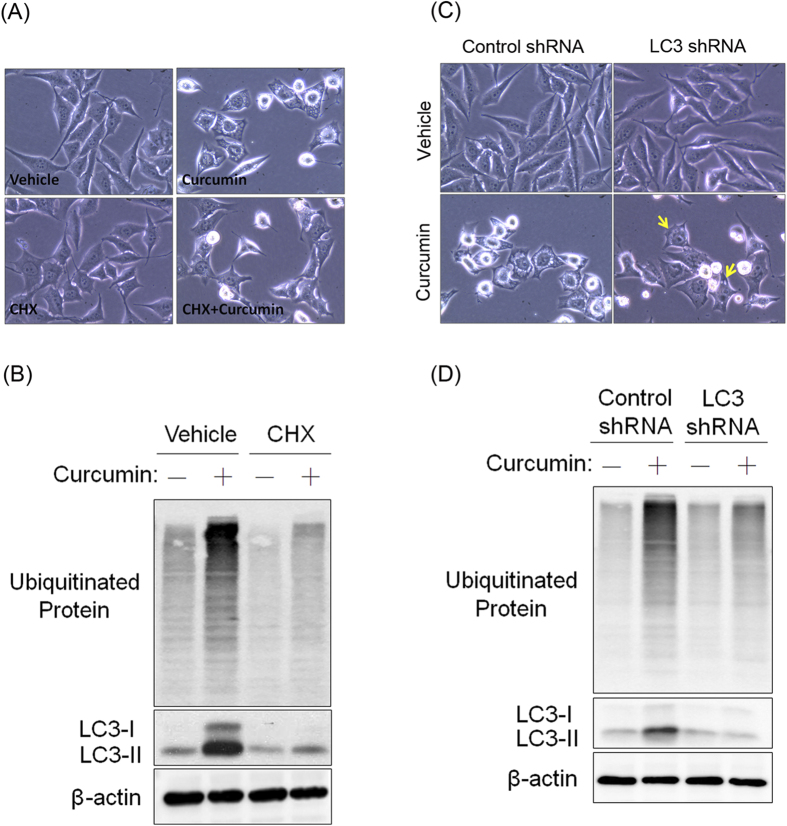
CHX and LC3 deficiency protect PC-3M cells from curcumin-induced cytoplasmic vacuolation-mediated cell death. (**A**): PC-3M cells were pretreated with CHX (25 μM) and then further treated with 50 μM curcumin for 24 h; then, phase-contrast images were observed. (**B**): Western blots showing the effect of CHX on the curcumin-induced expression of LC3 and ubiquitinated proteins. (**C**): Phase-contrast images showing the effect of LC3 knockdown on curcumin-induced cytoplasmic vacuolation. Vacuolated PC-3M cells are indicated by yellow arrows. (**D**): Western blots of the total cell lysates showing the effect of LC3 knockdown on the curcumin-induced expression of LC3 and ubiquitinated proteins.

Cytoplasmic vacuolation-mediated cell death and paraptosis have specifically been described to induce cytoplasmic vacuole formation, which is prevented by the protein translation inhibitor CHX^12^,^13^,^17^. However, silencing LC3 significantly protects against cytoplasmic vacuolation-mediated cell death^15^. To elucidate the mode of curcumin-induced cell death in PC-3M cells, we first blocked the biosynthesis of new proteins using CHX and then treated the PC-3M cells with curcumin. The cytoplasmic vacuolation induced by curcumin was completely blocked by CHX (Fig. 5A). The curcumin-mediated increases in ubiquitination and LC3 expression were also prevented by CHX (Fig. 5B). In addition to CHX, we observed that LC3 knockdown dominantly reversed curcumin-induced cytoplasmic vacuolation (Fig. 5C) and ubiquitinated protein accumulation (Fig. 5D). Taken together, our results indicated that cytoplasmic vacuolation-mediated cell death might be the major cause of the curcumin-induced death of PC-3M cells.

### Curcumin induces cytoplasmic vacuolation-mediated cell death by promoting an oxidative stress-mediated increase in ER stress in PC-3M cells

**Figure 6:**
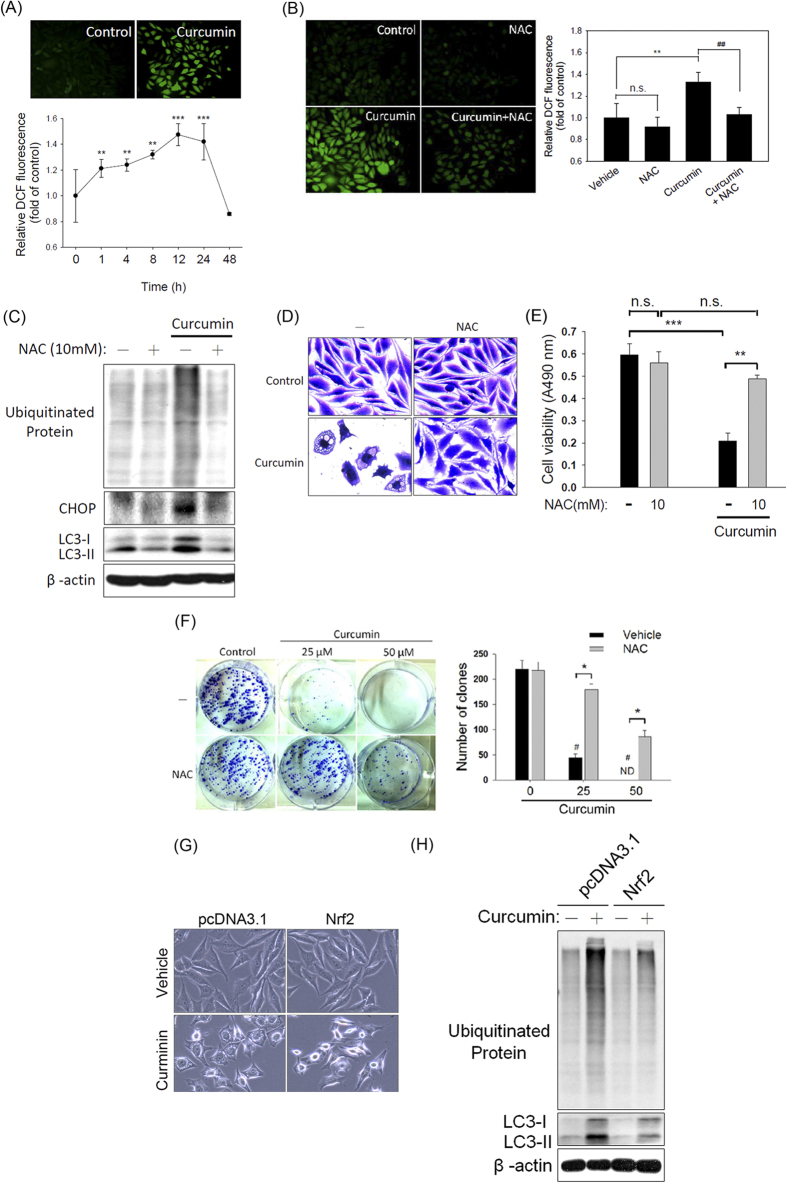
Oxidative stress plays a critical role in the curcumin-induced cytoplasmic vacuolation-mediated death of PC-3M cells. (**A**): PC-3M cells were treated with 50 μM curcumin for the indicated intervals (0-48 h) and stained with DCF-DA. The total ROS levels were then analyzed via spectrofluorophotometry. Top: Representative photomicrographs. Bottom: Quantification of ROS production as determined by DCF-DA fluorescence. Data are presented as the means ± SD. ** *P* < 0.01; *** *P* < 0.001 compared with the control. (**B**): Fluorescence microscopic images of PC-3M cells treated with 50 μM curcumin in the presence or absence of NAC. Left panel: Representative photomicrographs. Right panel: Quantification of ROS production according to DCF-DA fluorescence. Data are presented as the means ± SD. ** *P* < 0.01 compared with the control group; ^##^ *P* < 0.01 compared with the NAC and curcumin cotreatment group. (**C**): PC-3M cells were pretreated with 10 mM NAC for 1 h and subsequently treated with 50 μM curcumin for 24 h. After cell lysis, the expression of CHOP, LC3, and ubiquitinated proteins was analyzed via western blot. Equal protein loading was confirmed in the western blot by probing for β-actin. (**D**): Phase-contrast images of PC-3M cells treated with curcumin in the presence or absence of NAC for 48 h and then stained with crystal violet. (**E**): Cell viability was determined by the MTS assay. Data are presented as the means ± SD of 3 independent experiments. ** *P* < 0.01; *** *P* < 0.001. (**F**): Colony-forming assay. Left panel: Representative photomicrographs. Right panel: Data are presented as the means ± SD of 3 independent experiments. ^#^ *P* < 0.001 compared with the control group; * *P* < 0.001 compared with the NAC and curcumin cotreatment group. (**G**-**H**): Effect of Nrf-2 overexpression on the curcumin-induced cytoplasmic vacuolation (**G**) and expression of LC3 and ubiquitinated proteins (H).

Based on previous reports indicating that curcumin induces vacuolation or cell death by promoting the release of ROS^17^, we evaluated the possible involvement of ROS in curcumin-induced cytoplasmic vacuolation-mediated cell death. The cellular ROS levels were monitored using 2′,7′-dichlorofluorescein diacetate (DCF-DA), a fluorescent probe that is widely used to measure cellular ROS levels. We observed that the production of ROS was markedly increased after the PC-3M cells were treated with curcumin for 1 h and that this level of ROS production was maintained for 24 h (Fig. 6A). To more definitively determine whether increased intracellular ROS production is involved in the curcumin-mediated increase in ER stress in PC-3M cells, we pretreated the PC-3M cells with the antioxidant NAC and then exposed the cells to 50 μM curcumin for 1 h. The results showed that the increase in ROS production after a 1-h curcumin treatment was significantly attenuated by pretreating the cells with NAC (Fig. 6B). To further explore the importance of ROS production in the curcumin-induced increase in ER stress and subsequent vacuolation-mediated cell death, NAC was used to suppress curcumin-induced ER stress marker expression and vacuolation-mediated cell death. NAC pretreatment markedly reversed the curcumin-induced increase in the expression of ubiquitinated proteins, CHOP, and LC3 (Fig. 6C) and in cytoplasmic vacuolation (Fig. 6D). Moreover, NAC pretreatment significantly ameliorated the curcumin-mediated reduction in cell viability (Fig. 6E) and clonogenic survival (Fig. 6F).

NF-E2-related factor 2 (Nrf2) is a transcription factor that has been reported to regulate several antioxidant genes, such as glutathione peroxidase (GPx), NAD(P)H quinone oxidoreductase (NQO)-1, and heme oxygenase-1 (HO-1)^25^. To further confirm the role of oxidative stress in curcumin-mediated cytoplasmic vacuolation-mediated cell death, we overexpressed Nrf2 in PC-3M cells (Figure S1A) and evaluated the effect of Nrf-2 overexpression on curcumin-induced vacuolation and related protein expression (Fig. 6G,H). Gpx, NQO-1, and HO-1, the antioxidant genes downstream of Nrf2, were upregulated in the Nrf-2-overexpressing PC-3M cells (Figure S1B). Moreover, the overexpression of Nrf2 significantly prevented the curcumin-mediated increases in cytoplasmic vacuolation (Fig. 6G), ubiquitinated protein accumulation and LC3 expression (Fig. 6H). Overall, these results suggested that curcumin exposure is directly involved in oxidative stress induction leading to the ER stress-induced cytoplasmic vacuolation-mediated death of PC-3M cells.

### Curcumin suppresses tumor growth via oxidative stress-induced vacuolation-mediated cell death in the PC-3M orthotopic tumor model

**Figure 7:**
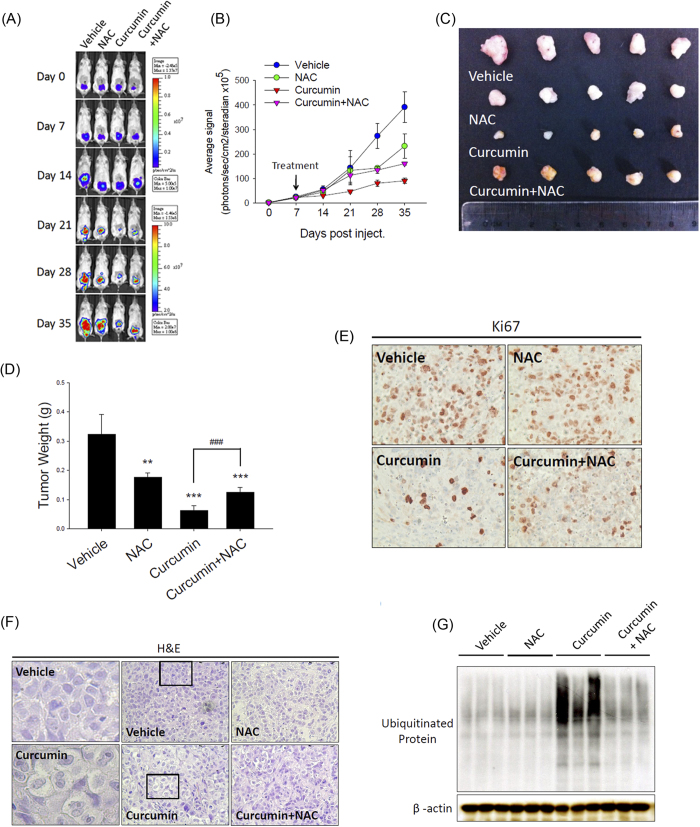
Curcumin suppresses tumor growth via oxidative-stress-induced vacuolation-mediated cell death in a PC-3M cell-based orthotopic tumor model. (**A**): Xenogen IVIS spectrum bioluminescence imaging of orthotopic prostate tumor growth. SCID mice were injected with PC-3M cells into the prostate and treated with curcumin (1.5 mg/mouse, intraperitoneal), curcumin and NAC (1 g/kg, oral), vehicle (control) or NAC alone. The mice were then subjected to Xenogen imaging at the indicated time points. (**B**): Quantitative analysis of the Xenogen imaging signal intensity (photons/sec/cm^2^/sr) over time after cell injection. (**C**): Representative imaging results from the indicated treatment protocols at 35 d after cancer cell injection are shown. (**D**): Relative weights of the tumors isolated from each group of tumor-bearing mice at the end of the experiment. (**E** and **F**): The tumor tissues were examined via immunohistochemical staining using the anti-Ki-67 antibody (**E**) or hematoxylin and eosin staining (left panel: magnification of the boxed areas in the vehicle- and curcumin-treated groups) (**F**). (**G**): The expression of ubiquitinated proteins in orthotopic tumors. Equal protein loading was confirmed in the western blot by probing for β-actin.

Based on the finding that increased ROS-mediated ER stress plays a critical role in curcumin-induced cytoplasmic vacuolation-mediated cell death *in vitro*, we next analyzed the *in vivo* antitumor effect of curcumin and the role of ROS accumulation in this effect. We established an orthotopic prostate tumor-bearing model by transplanting PC-3M cells into SCID mice. The PC-3M cell-xenografted mice were treated with curcumin (1.5 mg/mouse, intraperitoneal), curcumin+NAC (1 g/kg, oral), vehicle (control) or NAC alone each day for 4 wk beginning 7 d after tumor cell implantation. Tumor growth was monitored by observing and quantitatively analyzing the orthotopic bioluminescence imaging data from the luciferase-tagged tumors for 35 d using the IVIS imaging system. We found that the PC-3M-derived orthotopic prostate tumors of the mice receiving curcumin displayed a remarkable reduction in orthotopic bioluminescence, whereas the simultaneous administration of NAC and curcumin significantly ameliorated the effect of curcumin on the tumor burden (Fig. 7A,B). Representative tumor masses of the PC-3M cell-based xenografts from mice receiving the vehicle control, NAC alone, or curcumin with or without NAC showed that the tumor volume (Fig. 7C) and weight (Fig. 7D) in the curcumin-treated mice at 35 d were markedly reduced compared with those in the mice treated with vehicle or NAC alone. Moreover, NAC and curcumin cotreatment significantly reversed the antitumor effects of curcumin in this PC-3M orthotopic model (Fig. 7C,D). To determine whether curcumin exhibits antitumor activity *in vivo* by inhibiting cell proliferation and by inducing cytoplasmic vacuolation, proliferating cells were detected via immunocytochemical staining for Ki67, and vacuolation was evaluated via hematoxylin and eosin staining. After curcumin treatment, the number of Ki67-positive cells was reduced (Fig. 7E), but vacuolation in the tumor tissue appeared to increase (Fig. 7F) compared with the control treatment. Next, we measured the accumulation of ubiquitinated proteins in PC-3M cell-based xenografts harvested from the vehicle- or curcumin-treated mice and found that the ubiquitinated protein levels were increased by curcumin treatment and that this effect was reversed by cotreatment with NAC and curcumin (Fig. 7G). Overall, these results suggested that curcumin induces increased ER stress by enhancing intratumoral oxidative stress. This process may play a critical role in the curcumin-induced cytoplasmic vacuolation-mediated death of PC-3M cells *in vivo*.

## Discussion

Prostate cancer is the most common malignancy in men, and at present, no effective treatment for advanced prostate cancer has been developed. Despite recent advances in prostate cancer therapy, disease progression remains unavoidable, resulting in aggressive and metastatic tumors that are fatal. Thus, treatment resistance remains the major challenge for prostate cancer therapy^26^. Docetaxel is considered to be the most promising chemotherapeutic drug for prostate cancer treatment and causes significant cancer cell death by inducing apoptosis^27^. However, prostate cancer patients differ in terms of docetaxel resistance, which leads to disease relapse. Thus, the development of novel therapeutic approaches for prostate cancer treatment is required to restore the ability of chemotherapeutic agents to induce cell death and, thus, to improve therapeutic efficacy.

Obtaining a better understanding of the regulatory mechanisms that trigger forms of cell death other than apoptosis is crucial for eradicating prostate cancer. Our study showed the molecular signaling pathways underlying a unique form of cell death, cytoplasmic vacuolation-mediated cell death, which was induced by curcumin in androgen-independent and PTEN-null metastatic PC-3M cells. The major cytopathological characteristics of this type of cell death are cytoplasmic vacuolation predominantly resulting from ER stress, a lack of caspase activation, and increased protein ubiquitination^15^. Importantly, cytoplasmic vacuolation-mediated cell death has shown the ability to inhibit the growth of various therapy-resistant cancer types, such as apoptosis-resistant leukemia^12^, the most aggressive and treatment-resistant breast tumors, triple-negative breast cancer,^13^ and the castration-resistant prostate cancer cell line DU145^15^. These observations strongly suggest that the induction of cytoplasmic vacuolation-mediated cell death is a novel therapeutic strategy for treating drug-resistant cancers.

PC-3M cells were originally derived from liver metastases produced in nude mice following the intrasplenic injection of PC-3 cells. In our study, we observed curcumin-induced cytoplasmic vacuolation-mediated cell death in PC-3M cells but not in the parental PC-3 cells. One possible reason for these different responses to curcumin treatment might be that total Akt was expressed at similar levels between PC-3M and PC-3 cells but that the phospho-Akt level was higher in PC-3 cells than in PC-3M cells, suggesting the preferential activation of Akt in PC-3 cells compared to PC-3M cells^28^. The aberrant activation of Akt signaling prevents apoptosis, and the inhibition of the Akt signaling pathway has been reported to induce apoptosis in cancer cells^29^. Curcumin has been shown to inhibit cell proliferation, induce apoptosis and sensitize tumor cells to cancer therapies by inhibiting of Akt signaling in various tumor cell types^30^,^31^,^32^. Based on the difference in the endogenous level of phospho-Akt between PC-3M cells and PC-3 cells, PC-3M cells may weakly exhibit curcumin-induced apoptosis due to inhibition of the Akt pathway.

The present study showed that the anticancer activity of curcumin was distinct from autophagic and apoptotic processes, as indicated by the observation that neither the autophagy inhibitor 3-MA nor the apoptosis inhibitor z-VAD prevented the curcumin-induced cytoplasmic vacuolation-mediated death of PC-3M cells. A previous report indicated that manumycin A induces similar nonautophagic and nonapoptotic cell death pathways, along with LC3-regulated cytoplasmic vacuolation-mediated cell death, in triple-negative breast cancer^13^. LC3, a homolog of the yeast gene Atg8, has been found to be expressed in 2 major forms, the cytosolic type I (LC3-I) and the membrane-bound type II (LC3-II), which is derived from LC3-I via proteolysis and lipid modification^33^. When cells undergo autophagic cell death, it has been shown that LC3-I is processed into LC3-II to promote autophagosome formation, and the amount of LC3-II strongly correlates with the number of autophagosomes, suggesting that LC3 processing serves as an indicator of autophagy^34^. Another protein, Beclin 1, mediates the activation of autophagy and contains a short BH3 motif that binds to Bcl-2^35^. This interaction is responsible for autophagosome formation^36^. Therefore, changes in the levels of Beclin-1 and LC3-II may reflect the various stages of autophagy. In this study, we found that curcumin persistently induced cytoplasmic vacuolation and cell death by increasing LC3 activation but not by altering the expression levels of Beclin-1. These observations indicated that vacuoles were not efficiently fused to lysosomes and that the autophagic process remained incomplete in curcumin-induced cytoplasmic vacuolation-mediated cell death. We suggest that, similar to its role in manumycin-A-induced LC3-regulated vacuolation-mediated cell death^13^, LC3 may play a novel role in curcumin-induced cytoplasmic vacuolation-mediated death in PC-3M cells.

The ER is a cellular organelle in which secretory and membrane proteins are synthesized, modified, and folded. The ability of cells to respond to perturbations in ER functioning, including increases in protein synthesis and protein misfolding, is critical to the development of ER stress^37^. Moreover, changes in the expression of ER-stress-regulating proteins, such as Bip/GRP78 and CHOP, caused by the accumulation of unfolded proteins triggers the UPR^38^. Under normal conditions, unfolded proteins are ubiquitinated and degraded by the proteasome. The failure of chaperones to repair unfolded proteins results in the accumulation of these ubiquitinated proteins. This process has also been observed to be associated with ER-derived cytoplasmic vacuole formation and with enhanced antitumor activity^39^. Because the division rate of cancer cells is higher than that of normal cells, cancer cells accumulate more ubiquitinated proteins and are more sensitive to the death-inducing effects of proteasomal inhibitors than are normal cells. Based on the present findings, we suggest that the induction of ER stress and the accumulation of ubiquitinated proteins represent novel treatment strategies for cancer.

ROS constitute a crucial group of molecules that mediate numerous signal transduction pathways and perform critical functions in cells. Most cancer cells exhibit increased aerobic glycolysis and oxidative stress compared with those of their normal counterparts^40^. However, an increase in ROS production past a certain level may enhance the susceptibility of cancer cells to chemotherapeutic drugs. The present results showed that curcumin increases ROS production and subsequently induces ER stress, thus suggesting that the curcumin-mediated promotion of ER stress via ROS generation represents promising preventive and therapeutic strategies for prostate cancer. However, the mechanism by which ROS release leads to ER stress induction remains unclear. Recently, ROS have been reported to act as common early danger signals and to be a major source of ER stress. Indeed, a previous report has indicated that ROS generation is an early event that triggers ER stress via the ROS-dependent activation of the PERK–eIF2α–ATF4-mediated UPR^41^. In our study, we found that curcumin induced an ROS-mediated increase in ER stress, but the role of the ER kinase PERK in this process should be further investigated.

The current guidelines for prostate cancer treatment indicate that approximately 35% of patients experience disease recurrence within 10 years of primary therapy^42^. Developing a treatment for recurrent, late-stage, castration-resistant metastatic prostate cancer remains an unsolved therapeutic challenge. Thus, it is essential to develop a novel therapeutic strategy for advanced prostate cancer. In this study, we characterized the potential tumoricidal activity of curcumin by treating PC-3M cells with curcumin and examining its effects *in vitro* and *in vivo*. Our results indicate that curcumin induces a novel cytoplasmic vacuolation-mediated cell death pathway that is distinct from the apoptotic and autophagic cell death pathways. This novel pathway may prevent cancer cells from developing apoptosis or autophagy resistance after the completion of drug treatment. To further understand these mechanisms, we demonstrated that curcumin-induced ROS release is an initial signal for the induction of ER stress and for the simultaneous upregulation and processing of LC3, which subsequently increases ubiquitinated protein accumulation. Further investigation of the molecular mechanisms underlying curcumin-induced cytoplasmic vacuolation-mediated cell death may lead to the development of a novel therapeutic approach for the more effective management of prostate cancer.

## Methods

### Materials

Curcumin, AO, MDC, NAC, z-VAD, CHX, and DCF-DA were purchased from Sigma (St. Louis, MO, USA). The class III PI3K inhibitor 3-MA was obtained from Calbiochem (La Jolla, CA, USA). The CellTiter 96 AQueous One Solution Proliferation Assay System was purchased from Promega (Madison, WI, USA). Antibodies against PARP, caspase-3, p62, Beclin-1, CHOP, phospho-eIF2α (Ser51), IRE1-α, Bip/GRP78 and Nrf2 were purchased from Cell Signaling Technology (Beverly, MA, USA). Antibodies against LC3 (Sigma) were also used. The anti-β-actin, anti-ubiquitin, goat anti-rabbit and anti-mouse IgG antibodies were purchased from Santa Cruz Biotechnology (Santa Cruz, CA, USA).

### Cell lines and cell culture

The human prostate cancer cell lines PC-3, DU145, and LNCap were obtained from American Type Culture Collection (ATCC, Manassas, VA, USA). PC-3M, a highly metastatic cell line derived from a hepatic metastasis of PC-3 cells^14^, was provided by Dr. Min-Liang Kuo (Graduate Institute of Biochemical Sciences, College of Life Science, National Taiwan University). The PC-3 and PC-3M cells were cultured in minimal essential medium (MEM; Gibco BRL, Grand Island, NY, USA); the DU145 cells were cultured in Dulbecco’s modified Eagle’s medium (DMEM; Gibco BRL); and the LNCap cells were maintained in T-medium (Gibco BRL) supplemented with 10% FBS (Invitrogen) and 1% antibiotic-antimycotic (Invitrogen). Regular passaging was performed at 70%–80% confluence via trypsinization using 1 × trypsin and 0.05% EDTA followed by resuspension in complete medium. The cells were cultured and maintained at 37 °C in a 5% CO_2_ and 95% air atmosphere.

### Measurement of cell viability (MTS assay)

Cells (1 × 10^4^) were seeded into each well of a 96-well plate in 100 μL of tissue culture medium. After 24 h of incubation to allow the cells to adhere, the cells were treated with curcumin and incubated for an additional 48 h. Cell viability was then determined by performing the colorimetric MTS assay using the CellTiter 96 AQueous One Solution Proliferation Assay System (Promega). This assay measures the bioreduction of the tetrazolium compound MTS by intracellular dehydrogenases in the presence of the electron-coupling reagent phenazine methosulfate. MTS and phenazine methosulfate were added to the culture wells, and the mixture was incubated for 2 h at 37 °C. The absorbance, which is directly proportional to the number of viable cells in culture, was measured at 490 nm using a microplate reader. The percentage of cytotoxicity was calculated based on the loss of viable cells in the cultures.

### Preparation of total cell extracts and western blot analysis

The cells were washed with PBS containing zinc ions (1 mM) and then lysed in radioimmunoprecipitation assay buffer (50 mM Tris buffer, 5 mM EDTA, 150 mM NaCl, 1% NP40, 0.5% deoxycholic acid, 1 mM sodium orthovanadate, 81 μg/mL aprotinin, 170 μg/mL leupeptin, and 100 μg/mL PMSF; pH 7.5). After mixing for 30 min at 4 °C, the lysates were centrifuged (10^4^ × *g*) for 30 min, and the supernatants were collected as whole cell extracts. The protein content was determined using the Bio-Rad protein assay reagent with bovine serum albumin as a standard. Samples containing approximately 10–50 μg of protein were boiled in Laemmli sample buffer, separated on SDS-polyacrylamide gels, electrophoretically transferred to nitrocellulose membranes (Amersham, Arlington Heights, IL, USA), and probed with the indicated primary antibodies. The proteins were visualized using horseradish-peroxidase-conjugated secondary antibodies (Zymed Laboratory, South San Francisco, CA, USA) followed by chemiluminescence detection (ECL-Plus; Santa Cruz Biotechnology).

### MDC staining

For autophagic fluorescence observation, 2 × 10^4^ cells per well were seeded into 24-well plates and treated with 50 μM curcumin. After 24 h, the cells were stained with 0.05 mM MDC at 37 °C for 1 h. The changes in cellular fluorescence were observed under a fluorescence microscope (Zeiss Axioplan). As an autophagy control, the cells were serum-starved for 72 h.

### Detection and quantification of acidic vesicular organelles via AO staining

Autophagy is the process of sequestering cytoplasmic proteins into a lytic compartment and is characterized by the formation and accumulation of acidic vesicular organelles. To detect acidic cellular compartments, we used AO, which emits bright red fluorescence in acidic vesicles and green fluorescence in the cytoplasm and the nucleus. Cells (2 × 10^4^ per well) were seeded into 24-well plates and treated with 50 μM curcumin for 24 h. Subsequently, AO was added at a final concentration of 1 mg/mL for 15 min. Images were captured using a fluorescence microscope (Zeiss Axioplan). As an autophagy control, the cells were serum-starved for 72 h.

### Measurement of the intracellular ROS levels

The ROS levels were measured using the fluoroprobe DCF-DA (Sigma). Approximately 2 × 10^4^ cells per well were seeded into a 24-well plate, cultured in MEM containing 10% FBS, and treated with 50 μM curcumin in the absence or presence of NAC for the indicated intervals. After treatment, the cells were incubated in the dark in 50 μM DCF-DA for 30 min at 37 °C. Images were captured using a fluorescence microscope (Zeiss Axioplan). For the immunosorbent assay, the cells were resuspended in 500 μL of 0.2% SDS and transferred to a 96-well plate in duplicate. DCF-DA fluorescence was measured using a spectrophotometer (GENios Plus, TECAN) according to the manufacturer’s instructions. The fluorescence was normalized to the number of viable cells.

### Plate colony formation assay

PC-3M cells (1 × 10^3^ per well) were individually and concurrently seeded onto 6-well culture plates and then cultured under standard conditions for 2 wk. The medium was replaced every 3 d. Finally, the cells were stained with crystal violet, and colonies with more than 50 cells were counted. All experiments were performed in triplicate and repeated 3 times.

### DNA transfection

The Nrf2 plasmid was provided by Dr. Min-Liang Kuo (Graduate Institute of Biochemical Sciences, College of Life Science, National Taiwan University), and the shLC3 plasmid was provided by Dr. Shun-Fa Yang (Institute of Medicine, Chung Shan Medical University). For Nrf2 overexpression or LC3 knockdown, semiconfluent cultures of PC-3M cells in a 6-mm^2^ Petri dish were transfected with 5 μg of empty or expression vector (pcDNA3.1) using Lipofectamine Plus reagent. After incubation for 48 h, the cells were analyzed for the expression of exogenous Nrf2 or LC3 via western blot using an anti-Nrf2 or anti-LC3 antibody.

### TEM

The cells were prefixed in Karnovsky’s solution (1% paraformaldehyde, 2% glutaraldehyde, 2 mM calcium chloride, 0.1 M cacodylate buffer, pH 7.4) for 2 h and washed with cacodylate buffer. Post-fixing was performed using 1% osmium tetroxide and 1.5% potassium ferrocyanide for 1 h. After dehydration with 50–100% alcohol, the cells were embedded in Poly/Bed 812 resin (Pelco, Redding, CA, USA), polymerized, and observed under an electron microscope (EM 902A, Zeiss).

### RNA isolation and quantitative RT-PCR (qRT-PCR) analysis

Total RNA was isolated from PC-3M cells using the RNeasy kit (Qiagen, Valencia, CA, USA). A 2 μg sample of reverse-transcribed cDNA (Optimaz First Strand cDNA Synthesis Kit, Biochain, Newark, CA, USA) were amplified via qRT-PCR using 10 μM gene-specific primers and iQ SYBR Green Supermix (Bio-Rad). The expression level of 18S was used as an internal control. The following primers were used: GAPDH: 5′-GGA TGC AGG GAT GAT GTT C-3′ (sense) and 5′-TGC ACC ACC AAC TGC TTA G-3′ (antisense); Nrf2: 5′-CTC GCT GGA AAA AGA AGT G-3′ (sense) and 5′-CCG TCC AGG AGT TCA GAG G-3′ (antisense); and HO-1: 5′-TAA GAC CGC CTT CCT GCT CAA CAT-3′ (sense) and 5′-TGC TGG TTT CAA AGT TCA GGC CAC-3′ (antisense); NQO-1: 5′-TGG TTG TCA GTT GGG ATG GA-3′ (sense) and 5′-ATG GAA GAA ACG CCT GGA GA-3′ (antisense); GPx: 5′-CCC ACC AGG AAC TTC TCA AA-3′ (sense) and 5′-GAG CCC AAC TTC ATG CTC TT-3′ (antisense). The reaction conditions included an initial denaturation step at 95°C for 30 s, followed by 40 cycles of 95 °C for 5 s and 60 °C for 34 s; melting curve analysis was performed using the 7500 Real-Time PCR System (Applied Biosystems).

### Animal model

The PC-3M cells were cultured in MEM supplemented with 10% (v/v) fetal calf serum. For intraprostatic tumor growth, 5-week-old male SCID mice were anesthetized with pentobarbital, and approximately 5 × 10^5^ PC-3M cells were inoculated into the prostate with a 30-gauge needle, which was inserted through a lower abdominal incision. The incision was closed using a 4-0 Vicryl filament. The mice were sacrificed at various intervals after implantation and xenografting. Seven days after the injection, the mice were randomly assigned to the experimental and control groups according to the Xenogen IVIS spectrum bioluminescence imaging results such that the treatment was initiated at a similar mean tumor size for each group. Then each treated mouse received daily intraperitoneal injections of 1.5 mg of curcumin dissolved in DMSO. The injection volume was 100 μL (10 μL of a stock solution and 90 μL of PBS) each day. The control group received 100 μL of vehicle (10 μL of DMSO and 90 μL of PBS). The antioxidant-treated mice received drinking water supplemented with 40 mM NAC to yield an average dose of 1 g NAC/kg body weight/day, and the NAC-treated water was replaced every third day. All experiments were conducted in accordance with the guidelines and regulations approved by the Institutional Animal Care and Use Committee of Taipei Medical University with a reference number of LAC-2014-0128.

### Bioluminescence of prostate tumor cells

To track the tumor cells, luciferase-expressing PC-3M-Luc human prostate tumor cells were used. After luciferin injection, the same live imaging device (IVIS spectrum, Xenogen) was used to visualize and localize the bioluminescence.

### Immunohistochemistry

All tumor tissue samples were fixed in a buffered 10% formaldehyde solution. The specimens were embedded in paraffin blocks and sliced into 4-μm sections. All specimens were deparaffinized and immersed in 10 mM sodium citrate buffer (pH 6.0) in a microwave oven 2 times for 5 min for antigen retrieval. After washing, the slides were incubated in 0.3% H_2_O_2_ in methanol to quench the endogenous peroxidase activity. The slides were washed with PBS and incubated in the anti-Ki67 antibody at a 1:100 dilution for 1 h at room temperature. After washing with PBS, the slides were developed using a Vectastain avidin–biotin complex (ABC) peroxidase kit (Vector Laboratories, Burlingame, CA, USA) and a 3,3,9-diaminobenzidine peroxidase substrate kit (Vector Laboratories) according to the manufacturer’s instructions. All of the specimens were deparaffinized and stained with hematoxylin and eosin, which were used as a light counterstain.

### Statistical analysis

Data points represent the mean ± SD. Data comparisons between 2 groups were analyzed using Student’s *t-*test. One-way ANOVA followed by the Tukey post hoc test was used to evaluate differences between 3 or more groups. The differences were considered to be significant at a 95% confidence interval (*P* < 0.05).

## Additional Information

**How to cite this article**: Lee, W.-J. *et al.* Nonautophagic cytoplasmic vacuolation death induction in human PC-3M prostate cancer by curcumin through reactive oxygen species -mediated endoplasmic reticulum stress. *Sci. Rep.* **5**, 10420; doi: 10.1038/srep10420 (2015).

## Supplementary Material

Supporting Information
