# Corrigendum: Nonautophagic cytoplasmic vacuolation death induction in human PC-3M prostate cancer by curcumin through reactive oxygen species -mediated endoplasmic reticulum stress

**DOI:** 10.1038/srep42453

**Published:** 2017-02-22

**Authors:** Wei-Jiunn Lee, Ming-Hsien Chien, Jyh-Ming Chow, Junn-Liang Chang, Yu-Ching Wen, Yung-Wei Lin, Chao-Wen Cheng, Gi-Ming Lai, Michael Hsiao, Liang-Ming Lee

Scientific Reports 5: Article number: 1042010.1038/srep10420; published online: 05 27 2015; updated on: 02 22 2017

In this Article, Gi-Ming Lai is incorrectly listed as being affiliated with ‘Division of Hematology and Medical Oncology, Department of Internal Medicine, Wan Fang Hospital, Taipei Medical University, Taipei, Taiwan’. The correct affiliation is listed below:

Department of Internal Medicine, School of Medicine, College of Medicine, Taipei Medical University, Taipei, Taiwan

