# Supplementary material for: Nonautophagic cytoplasmic vacuolation death induction in human PC-3M prostate cancer by curcumin through reactive oxygen species -mediated endoplasmic reticulum stress
Source: Sci Rep. 2015 May 27;5:10420. doi: 10.1038/srep10420 (PMC4445067; doi:10.1038/srep10420)
Supplement: Supporting Information [file srep10420-s1.pdf]

Supplementary Information

Nonautophagic cytoplasmic vacuolation death induction in human PC-3M prostate cancer by curcumin through reactive oxygen species-mediated endoplasmic reticulum stress

Wei-Jiunn Lee[#], Ming-Hsien Chien[#], Jyh-Ming Chow, Junn-Liang Chang, Yu-Ching Wen, Yung-Wei Lin, Chao-Wen Cheng, Gi-Ming Lai, Michael Hsiao & Liang-Ming Lee[*]

Supplementary Figure S1

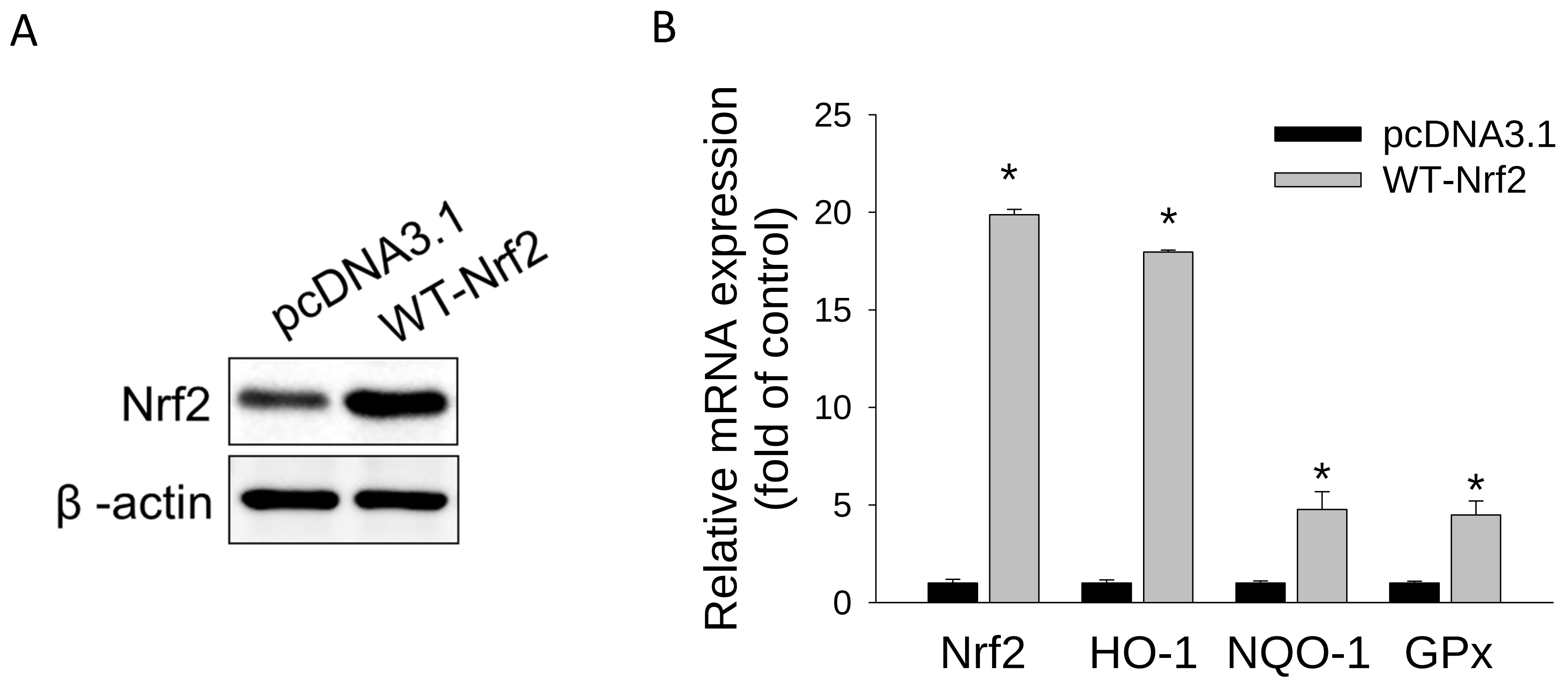

Figure S1. Functional confirmation of Nrf2 expression in PC-3M cells. (A): PC-3M cells were transfected with Nrf2 or a vector control (pcDNA 3.1), and expression levels of Nrf2 were measured by a Western blot analysis. (B): RNA was respectively isolated from PC-3M/vector and PC-3M/Nrf2 and applied to a real-time polymerase chain reaction assay to detect expression levels of Nrf2 and several Nrf-2-regulated genes: heme oxygenase (HO)-1; NAD(P)H quinone oxidoreductase (NQO)-1; and glutathione peroxidase (Gpx). The results are expressed as the means ± SD of three independent experiments. The asterisks indicate significant differences compared to controls ($p < 0.01$).
